# Multidimensional structure, measurement of patient capital, and its impact on team innovation performance

**DOI:** 10.3389/fpsyg.2026.1691386

**Published:** 2026-06-10

**Authors:** Risheng Qiao, Yongsheng Qiao, Yongmei Qiao

**Affiliations:** 1 School of Finance and Economics, Taiyuan University of Technology, Taiyuan, China; 2 Department of Management, Taiyuan University, Taiyuan, China; 3 Foreign Affairs Service Center, Mentougou District Government of Beijing Municipality, Beijing, China

**Keywords:** patient capital, team innovation performance, grounded theory, transformational leadership, MSEM

## Abstract

**Introduction:**

This study aims to explore the multidimensional structure of patient capital, develop a standardized measurement tool, and reveal its mechanism of influence on team innovation performance.

**Methods:**

Based on classical grounded theory, NVivo 12 software was used to conduct a three-stage coding analysis of qualitative research data. Through exploratory and confirmatory factor analyses, a second-order five-factor measurement model of patient capital was established, and its reliability and validity were verified via large‑scale surveys. Finally, a multilevel structural equation model (MSEM) was employed to examine the cross-level mediating role of patient capital in the relationship between transformational leadership and team innovation performance.

**Results:**

Five core dimensions of patient capital were identified: self-control and patience, persistence and resilience, long-term goal drive, emotional intelligence and interpersonal relationships, and adaptability and flexibility. The findings indicate that patient capital plays a significant mediating role in the relationship between transformational leadership and team innovation performance.

**Discussion:**

The results suggest that transformational leadership contributes to the cultivation of patient capital, which in turn is positively associated with enhanced team innovation performance. This study provides a validated measurement tool for patient capital and reveals its cross‑level mediating mechanism in organizational contexts.

## 1 Introduction

With the acceleration of globalization and the rapid advancement of technology, enterprises are facing an increasingly complex and uncertain environment. Against this backdrop, the concept of patience has gradually garnered widespread attention across interdisciplinary fields. As stated in the *Tao Te Ching*, “Great deeds are made up of small acts.” This ancient wisdom profoundly reveals the fundamental role of patience in achieving extraordinary undertakings, aligning with the philosophy of long-term investment and accumulation emphasized by patient capital. This insight appears even more prescient amid the rapid changes in today’s business environment and implies the importance of patient capital in business strategy.

Existing research has predominantly relied on financial indicators (such as investment cycles) to measure patient capital (Fichtner and Heemskerk, 2020), neglecting its cognitive and behavioral dimensions (e.g., managers’ time preferences, employees’ emotional self-control abilities, etc.). In the field of organizational management, patient capital is not only an important psychological resource influencing individual and team behavior but also a core driver promoting efficient teamwork, innovation, and sustained performance (Trudeau, 2018). Traditional human resource management theories have primarily focused on short-term performance, motivation, and incentives, with relatively little exploration of long-term goal orientation and emotional management. As the work environment becomes increasingly complex, corporate leaders and managers are gradually realizing that employees’ patience and persistence play an increasingly important role in advancing long-term projects, cross-departmental collaboration, and personal career development (Jiang and Street, 2019; Qiao and Qiao, 2024). Particularly in the Chinese context, the formation mechanism of patient capital and its role within organizations urgently require in-depth research.

Existing literature still lacks systematic theoretical construction and in-depth empirical research on patient capital within organizations—such as long-term incentive design and strategic resource commitment. In particular, the multidimensional structure of the concept of patient capital has not been fully theoretically explored or comprehensively measured. Moreover, cross-level studies (individual-team-organization) are relatively scarce, leaving the dynamic transmission pathways of patient capital insufficiently revealed. Therefore, exploring and measuring employees’ patient capital holds significant practical importance for optimizing corporate human resource management, improving team collaboration efficiency, and addressing future uncertainties.

Based on this, this study employs systematic theoretical analysis and empirical research to deeply explore the connotation, dimensions, and mechanism of patient capital’s impact on team innovation performance. First, grounded theory is used to inductively refine the multiple dimensions of patient capital. During the coding and comparison process, a close correlation between transformational leadership and patient capital was identified, preliminarily indicating that transformational leadership behaviors and styles positively influence the formation and development of patient capital. Subsequently, through a paired survey of 67 teams across 6 enterprises (involving 624 employees), a multilevel structural equation model is applied to validate the cross-level transmission mechanism of patient capital on team innovation performance. Finally, management recommendations are proposed for cultivating patient capital within organizations.

## 2 Grounded theory analysis

### 2.1 Sample selection and data collection

We employed a theoretical sampling strategy to select participants with substantial long-term work experience in management or R&D roles, ensuring the richness and theoretical saturation of the data. Participants were current EMBA and MBA students from Nankai University, Tianjin University of Technology, and Taiyuan University of Technology, chosen for their relevant professional backgrounds and ability to provide contextually grounded insights. Screening was based on respondents’ demonstrated understanding of and experience with long-term investment, sustained support, and innovation cultivation. Specifically, we selected those who could articulate concrete examples of pursuing long-term goals, fostering a failure-tolerant culture, and providing patient support during team challenges. A total of 45 interviewees were selected for the qualitative phase.

For the quantitative phase, we surveyed 67 R&D teams from six high-tech parks in China, comprising 624 employees. Team leaders and senior managers provided ratings of team innovation performance, while employees self-reported transformational leadership and patient capital.

During the sample screening process, respondents were selected based on their answers to questions related to patient capital, focusing on those who highly identified with the concept of patient capital and had practical operational experience. The main screening criteria included: whether the respondents demonstrated a deep understanding and emphasis on long-term investment, stable support, and innovation incentives in their work practices, particularly focusing on whether they consistently pursued long-term goals rather than merely emphasizing short-term outcomes. Additionally, respondents needed to specifically describe actual practices within their organizations regarding leadership support, opportunities for employee growth, and tolerance for failure, assessing whether their organizations fostered a cultural environment conducive to the accumulation of patient capital and innovative activities. Respondents were also expected to share concrete examples of the roles they played and the support they provided during team innovation processes, especially in how they demonstrated patience and offered necessary support to team members when facing challenges and difficulties.

### 2.2 Interview question design

After selecting 45 interviewees, a series of interview questions were designed to deeply explore the respondents’ understanding and application of patient capital in their actual work. To ensure the scientific rigor and objectivity of the interview process, five experts with extensive experience in organizational behavior and human resource management were invited to provide professional feedback and suggestions on the interview outline design and data analysis. The interview questions were designed based on the multidimensional structure of patient capital, covering its core components. Specific questions included: “In your work, do you emphasize the investment of long-term resources and time to promote the sustained development of projects? How do you implement this specifically?” “Does your organization encourage a culture that tolerates failure and supports long-term employee growth? Can you share some specific cases related to this?” “Have you ever encountered high-pressure or complex situations in your work? How did you adjust your emotions and behavior to maintain an efficient working state?” “In the process of achieving long-term goals, how do you maintain patience and avoid being distracted by short-term difficulties or outcomes? Can you share an experience where you persevered to achieve a long-term strategic goal?”

These questions were intended to guide respondents to share specific contexts and personal experiences, helping to deepen the understanding of the multidimensional characteristics of patient capital and its concrete operation in management practices.

### 2.3 Initial coding

In accordance with the requirements of open coding, NVivo 12 software was used to conduct a sentence-by-sentence and line-by-line analysis of all original interview content. Relevant concepts were extracted and categorized into different domains to complete the preliminary screening. Ultimately, 624 initial concepts were refined (specific examples are shown in Table 1).

**Table 1:** Examples of initial coding.

Code	Content description	Initial coding
A1-2	Able to self-regulate and remain calm in the face of unexpected situations, helping stabilize team emotions.	Emotional self-control: ability to remain calm under pressure and manage emotions.
A1-3	When the team encounters setbacks, able to learn from failures, quickly adjust strategies, and not give up easily.	Resilience and recovery: ability to recover from failure, persist in efforts, and not abandon goals.
A1-4	Set a two-year career development goal and demonstrated strong long-term goal orientation despite repeated challenges.	Long-term goal drive: setting and adhering to long-term goals with patience.
A1-5	Demonstrated strong empathy during team discussions, listening to and understanding differing opinions, and patiently explaining own views.	Empathy: ability to understand others’ perspectives and emotions, and to listen patiently.
A1-6	Exhibited high self-motivation in tasks, maintaining efficiency even when facing difficulties.	Self-motivation: maintaining drive and focus in the face of challenges.
A1-7	Remained calm during task execution, engaged in careful planning, avoided hasty actions, and continuously adjusted methods.	Calm decision-making and planning: Avoiding impulsive decisions; engaging in patient long-term planning.
A1-8	Demonstrated patience in resolving team conflicts, proactively mediating to ensure consensus with a positive attitude.	Conflict mediation: patience in handling team conflicts and promoting cooperation.
A1-9	When taking on new projects, despite new challenges, showed strong adaptability and quickly adjusted work strategies.	Adaptability and flexibility: ability to rapidly adapt to new tasks and environments and adjust strategies.
A1-10	Regular self-reflection in daily work to assess personal growth and ensure actions align with goals.	Self-reflection: regular self-evaluation and reflection to ensure self-improvement and goal alignment.
A1-11	Demonstrated considerable patience in handling complex tasks, solving problems step by step, and ultimately achieving objectives.	Patient execution: showing patience in complex tasks, progressing steadily to completion.
A1-12	Tended to proactively understand team members’ needs and supported their growth with continuous encouragement.	Team support and encouragement: actively supporting team members, demonstrating empathy and motivational ability.

### 2.4 Selective coding

Through in-depth analysis, screening, categorization, and integration of the concepts extracted during the initial coding phase, 15 frequently occurring and highly correlated subcategories were identified. These were further consolidated into 5 core categories. Examples are provided in Table 2.

**Table 2:** Examples of selective coding.

Core category	Subcategory	Open codes
Self-control and patience	Emotional regulation	A1-3 Able to control emotional reactions and avoid anxiety or impatience; A2-5 Remaining calm under pressure and avoiding impulsive decisions; A3-4 Managing emotional fluctuations and maintaining rationality.
	Calm decision-making	A4-2 Remaining calm when making decisions and avoiding emotional reactions; A1-7 Making rational judgments and decisions when facing challenges; A5-1 Avoiding rushed actions and considering long-term consequences.
	Self-control	A2-10 Remaining calm and avoiding overreaction in difficult situations; A3-11 Refraining from emotional responses to setbacks and maintaining patience; A1-6 Preventing emotions from affecting work efficiency.
Persistence and resilience	Persistence amid challenges	A2-4 Persisting in efforts without giving up when facing work difficulties; A1-6 Persevering and overcoming obstacles despite setbacks; A4-8 Continuing to strive toward goals after failure.
	Recovery from failure	A5-3 Quickly adjusting mindset after setbacks and moving forward; A2-12 Learning from failure and trying again; A3-7 Maintaining motivation and re-evaluating direction when encountering difficulties.
	Stress resilience	A5-9 Maintaining focus and continued effort in high-pressure environments; A1-10 Positively facing challenges even in tense situations; A4-6 Adjusting effectively to avoid excessive anxiety.
Long-term goal drive and self-motivation	Goal setting and persistence	A1-8 Setting long-term goals and persevering toward them; A2-9 Regularly evaluating and adjusting goals to maintain motivation; A4-6 Maintaining patience and not abandoning goals due to short-term difficulties.
	Self-motivation and encouragement	A2-11 Self-motivating even in the absence of external incentives; A3-5 Sustaining intrinsic motivation during fatigue; A5-4 Maintaining consistent work passion and not giving up easily.
	Goal and achievement accumulation	A4-9 Gradually accumulating achievements while pursuing long-term goals; A1-4 Transforming small successes into motivation to advance toward goals; A5-5 Gaining increased confidence in goals with each minor success.
Emotional intelligence and interpersonal relationships	Empathy and understanding	A2-7 Understanding others’ emotions and needs, respecting different perspectives; A3-5 Actively listening to others’ opinions, understanding and providing feedback; A5-8 Showing care and support for others, helping them grow.
	Communication and patient listening	A4-5 Excelling in communication with others, listening and responding patiently; A1-13 Respecting others’ viewpoints and demonstrating patience in communication; A3-8 Waiting patiently for others to express their opinions in discussions.
	Team relationship management	A4-11 Handling complex team relationships patiently and maintaining harmony; A1-9 Maintaining open and respectful relationships with colleagues to avoid conflict; A2-10 Willingly providing support and assistance to team members.
Adaptability and flexibility	Adaptive response to change	A2-13 Adapting quickly to changes in organizational or market environments; A4-5 Maintaining an open mindset and flexibly adjusting strategies in new environments; A1-10 Responding promptly to changes and adjusting course of action.
	Continuous learning and adjustment	A3-10 Maintaining a learning attitude and adapting quickly to new tasks; A5-4 Welcoming new challenges and adjusting work methods; A2-11 Remaining patient with new situations, learning and gradually mastering them.
	Flexible response to challenges	A1-12 Flexibly adjusting approaches when facing unknown tasks or challenges; A2-6 Adapting strategies based on actual conditions to maintain flexibility; A5-7 Remaining calm under pressure, adapting and solving problems.

### 2.5 Theoretical coding

The core objective of theoretical coding is to integrate the outcomes of the initial and selective coding stages to construct a systematic theoretical framework, thereby revealing the dynamic relationships among core categories and their synergistic mechanisms within organizational management.

#### 2.5.1 Relationships among core categories

Throughout the three-phase coding process of grounded theory, this study compared coded data from different leadership styles and identified a high degree of alignment between the four core dimensions of transformational leadership (Idealized Influence, Inspirational Motivation, Intellectual Stimulation, and Individualized Consideration) and the five dimensions of patient capital. The results are presented in Table 3.

**Table 3:** Dimensional alignment between transformational leadership and patient capital.

Transformational leadership	Patient capital	Mechanism pathway	Theoretical support
Idealized influence	Long-term goal drive	By conveying a clear long-term vision, leaders inspire individuals’ identification with organizational strategy, internalizing it into personal goal commitment and driving sustained pursuit of long-term objectives.	Transformational Leadership Theory: Idealized influence fosters employees’ identification with and commitment to the organizational vision, motivating them to pursue ambitious goals and thereby facilitating long-term goal orientation.
Idealized influence	Persistence and resilience	Leaders exemplify persistence and resilience through role modeling, inspiring members to maintain patience and perseverance when confronting challenges.	Social Learning Theory: Individuals develop similar emotional and behavioral traits by observing and imitating leaders’ behaviors. Idealized influence promotes members’ persistence and resilience through exemplary conduct.
Inspirational motivation	Adaptability and flexibility	By employing symbolic rewards (e.g., “innovation milestone recognition”) and positive feedback, leaders enhance members’ confidence and motivation for innovation, encouraging flexible adaptation to change and willingness to experiment with new approaches.	Motivation Theory: Intrinsic motivation and positive feedback strengthen individuals’ autonomy, thereby incentivizing adaptive behaviors.
Inspirational motivation	Persistence and resilience	Through positive incentives and recognition, leaders reinforce members’ tolerance for adversity, enabling them to maintain a positive attitude and persist without giving up when encountering difficulties.	Expectancy Theory: Incentive mechanisms influence individuals’ psychological responses to challenges. Inspirational motivation enhances members’ resilience and persistence through positive reinforcement, encouraging sustained effort in the face of difficulties.
Intellectual stimulation	Adaptability and flexibility	By encouraging members to challenge the status quo and promoting a culture of trial and iteration, leaders facilitate dynamic strategy adjustment and optimization, thereby improving the team’s adaptability and flexibility.	Transformational Leadership Theory: Intellectual stimulation activates individuals’ creative thinking and prompts continuous reflection and adjustment, thereby enhancing the team’s capacity to adapt to change.
Intellectual stimulation	Emotional intelligence and interpersonal relationships	By stimulating members’ intellectual vitality and encouraging open communication and collaboration, leaders cultivate emotional intelligence and interpersonal skills, enabling members to better manage relationships during innovation processes.	Emotional Intelligence Theory: Through intellectual stimulation, leaders not only promote cognitive innovation within the team but also enhance members’ emotional awareness and interpersonal management capabilities, thereby improving team collaboration and innovation.
Individualized consideration	Emotional intelligence and interpersonal relationships	By identifying and addressing members’ emotional needs, providing psychological support and a sense of security, leaders reduce conflict and pressure during innovation processes, promoting team harmony and collaboration.	Social Support Theory: Emotional support and individualized consideration help individuals maintain emotional stability in high-pressure environments, improving their emotional intelligence and interpersonal management capabilities, thereby enhancing team collaboration.

Transformational leadership motivates employees to pursue higher goals, exceed their own limitations, and maintain firm determination in the face of adversity, thereby facilitating the accumulation and transmission of patient capital and providing support for collaborative innovation at the team level. The five dimensions of patient capital—self-control and patience, persistence and resilience, long-term goal drive, emotional intelligence and interpersonal relationships, and adaptability and flexibility—reflect the psychological and behavioral traits required for employees to confront challenges and uncertainties. These dimensional traits not only help employees maintain patience amid short-term difficulties but also provide intrinsic motivation for long-term goal orientation. Guided by transformational leadership, patient capital at the individual level gradually aggregates to the team level, forming a “patience–innovation” positive feedback loop. This process not only reveals the transmission pathway of patient capital within teams but also highlights the critical role of transformational leadership in fostering team patience and innovation.

#### 2.5.2 Comparison of leadership styles

To compare the relationships between different leadership styles and patient capital, and to clarify why transformational leadership is particularly aligned with patient capital, five experts and practitioners from the fields of organizational behavior, leadership studies, and business management were invited to conduct an evaluation. To ensure objectivity and rationality in the scoring process, the following criteria were applied:

① Self-control and patience: whether the leadership style effectively promotes employees’ emotional regulation and ability to delay immediate gratification.
② Persistence and resilience: the influence of the leadership style on employees’ capacity to recover from adversity and sustain effort.
③ Long-term goal drive: the extent to which the leadership style facilitates employees’ ability to set and achieve long-term goals.
④ Emotional intelligence and interpersonal relationships: the impact of the leadership style on employees’ emotional intelligence, empathy, communication skills, and team coordination abilities.
⑤ Adaptability and flexibility: how the leadership style influences employees’ ability to respond to environmental changes and engage in dynamic learning and adjustment.

A 10-point scoring system was used, with 8–10 indicating high alignment, 4–7 partial alignment, and 1–3 low alignment. Weighted average calculations were performed on the experts’ ratings to determine the final alignment score between each leadership style and each dimension of patient capital. The results are presented in Table 4.

**Table 4:** Alignment scores between different leadership styles and dimensions of patient capital.

Dimension	Transformational leadership	Transactional leadership	Charismatic leadership	Ethical leadership	Servant leadership	Participative leadership
Self-control and patience	✔ High alignment	△ Partial alignment	✔ High alignment	✔ High alignment	△ Partial alignment	△ Partial alignment
Persistence and resilience	✔ High alignment	✔ High alignment	✔ High alignment	✔ High alignment	✔ High alignment	✔ High alignment
Long-term goal drive	✔ High alignment	⨯ Low alignment	△ Partial alignment	✔ High alignment	△ Partial alignment	✔ High Alignment
Emotional intelligence & relationships	✔ High alignment	✔ High alignment	✔ High alignment	✔ High alignment	✔ High alignment	✔ High alignment
Adaptability and flexibility	✔ High alignment	✔ High alignment	△ Partial alignment	△ Partial alignment	✔ High alignment	✔ High alignment

#### 2.5.3 Theoretical saturation test

To verify theoretical saturation, an in-depth exploration and validation were conducted using extensive additional literature after constructing the grounded theory framework. This step aimed to ensure that the theoretical framework comprehensively covered the main aspects of the studied phenomenon and that no significant omissions or unexplored dimensions remained.

First, a systematic review of literature related to the research topic was performed, comparing and analyzing perspectives and theoretical frameworks from existing studies against the grounded theory developed in this research. Second, multiple experts and peers with relevant disciplinary backgrounds were invited to independently code the literature, and their coding results were compared. Discrepancies were resolved through discussion and negotiation, further enhancing the reliability and validity of the theoretical framework. Finally, additional literature, particularly studies closely related to the phenomenon but not previously included, was collected and analyzed. These new materials were compared with the constructed theoretical framework to determine whether new dimensions or concepts needed to be incorporated.

As shown in Table 5, after completing the above validation steps, no new dimensions or concepts requiring integration into the theoretical framework were identified. This indicates that the constructed grounded theory framework has achieved a high degree of saturation and stability, and is capable of comprehensively reflecting the main characteristics and patterns of the studied phenomenon.

**Table 5:** Examples of theoretical saturation validation.

Dimension of patient capital	Literature comparison	Organizational behavior case comparison
Self-control and patience	Concepts of self-control and delayed gratification originate from psychological delayed gratification experiments; in Confucian thought, “endurance” is regarded as a key virtue in moral cultivation.	Google’s “20% Time Policy” allowed employees to demonstrate self-control by integrating long-term personal goals with daily innovation work, leading to outcomes such as the creation of Gmail.
Persistence and resilience	The spirit of “perseverance” and “persistence”; learned helplessness theory indicates that resilience is key to maintaining a positive attitude and capacity for action in adversity.	Sany Heavy Industry’s “Long March Initiative” incorporated resilience training into compulsory courses for engineers, cultivating persistence and resilience in the face of challenges and enhancing overall team response capabilities.
Long-term goal drive	The ancient admonition: “A journey of a thousand miles begins with a single step”; self-determination theory emphasizes that long-term goal orientation stimulates intrinsic motivation.	Tencent achieved phased goals focusing on long-term user growth and technological accumulation, expanding from social platforms to sectors such as payment and gaming, forming a sustained competitive advantage.
Emotional intelligence & interpersonal relationships	Emotional intelligence theory highlights the role of emotional management and interpersonal communication in promoting team collaboration. High emotional intelligence improves team coordination and conflict resolution.	Haier promoted emotional intelligence, fostering mutual growth between the enterprise and employees, thereby enhancing overall collaborative efficiency.
Adaptability and flexibility	Adaptation theory posits that adaptability is critical for the survival and development of organizations and individuals in rapidly changing environments.	ByteDance demonstrated remarkable adaptability and flexibility through agile market responses and product iterations, rapidly expanding from short-video platforms into sectors such as news and social networking.

#### 2.5.4 Definition of patient capital

Based on the above theoretical coding and comparative analysis, and integrating theories such as self-determination theory, emotional regulation theory, and social learning theory (Cohen and Wills, 1985; Deci and Ryan, 2000), patient capital is defined within the framework of organizational behavior as a long-term-oriented psychological and emotional resource. It manifests as an individual’s ability to sustain effort, maintain emotional stability, and remain driven by long-term goals when confronting challenges, pressures, and uncertainties, thereby demonstrating continuous effort, resilience, and adaptability in achieving both organizational strategic objectives and personal career development.

Patient capital can be categorized into five core dimensions:

*Self-control and patience:* the ability to regulate emotions and postpone immediate gratification in high-pressure and challenging situations.
*Persistence and resilience:* the capacity to withstand difficulties and adversity while maintaining sustained effort and a positive attitude.
*Long-term goal drive:* a strategic, forward-looking perspective and the ability to delay gratification in pursuit of overarching objectives.
*Emotional intelligence and interpersonal relationships:* abilities such as empathy, effective communication, and teamwork.
*Adaptability and flexibility:* the capability to learn, adjust, and innovate within dynamically changing environments.
*Distinction from existing constructs:* while patient capital shares conceptual space with established constructs such as Psychological Capital (PsyCap; e.g., self-efficacy, hope) and Grit (i.e., perseverance and passion for long-term goals), it offers distinct theoretical and practical value. First, patient capital is inherently time-centric and context-sensitive, focusing specifically on the sustained deployment of psychological resources in the face of innovation-related uncertainties and extended project lifecycles. Unlike the broader, more generalized positive appraisal of PsyCap, patient capital is activated under conditions of prolonged ambiguity and delayed returns. Second, patient capital uniquely integrates adaptive flexibility as a core dimension. This contrasts with the trait-like stability emphasized in Grit, positing instead that effective long-term persistence requires not just unwavering consistency but also the capacity for dynamic learning and strategic adjustment. Third, patient capital is theorized as a multi-level emergent resource. Our framework explicitly models the cross-level pathway from individual psychological attributes to collective team outcomes, whereas PsyCap and Grit are predominantly conceptualized and measured as individual-level constructs. These distinctions position patient capital as a complementary yet independent resource critical for understanding performance in innovation-driven, high-uncertainty organizational contexts.

### 2.6 Theoretical hypotheses

Building on the grounded theory analysis, with a focus on the relationship between transformational leadership and patient capital, the following theoretical hypotheses are proposed:

Transformational leadership, through idealized influence, communicates organizational vision and goals, thereby inspiring individuals’ commitment to and confidence in long-term objectives (Bass, 1990). This influence encourages individuals to demonstrate greater persistence and patience when confronting challenges. Furthermore, transformational leadership’s inspirational motivation enhances individuals’ resilience and adaptability in adverse situations through symbolic rewards and incentives, further accumulating patient capital. Additionally, individualized consideration addresses emotional needs and provides psychological safety, reducing emotional conflicts during innovation processes and sustaining long-term innovative motivation and patience. Thus, it is hypothesized:

*H1:* Transformational leadership has a positive effect on patient capital.

Patient capital reflects an individual’s resilience, long-term goal orientation, and adaptive flexibility when facing challenges. Individuals with higher levels of patient capital can maintain stable motivation and endurance throughout prolonged innovation processes, enabling continuous adjustment and improvement, especially in the face of failure or iteration. According to psychological capital theory, patient capital helps individuals effectively cope with pressure and sustain resource investment, facilitating the generation and implementation of innovative thinking (Bandura, 1977). Moreover, through collaborative mechanisms, patient capital can flow and interact among team members, enhancing the team’s overall innovative capability and performance. Thus, it is hypothesized:

*H2:* Patient capital has a positive effect on team innovation performance.

Furthermore, based on social exchange theory and multi-level emergence theory (Gross, 2015), transformational leadership stimulates individual-level patient capital, thereby promoting a stable and sustained environment for team innovation. Patient capital at the individual level aggregates at the team level, forming a collective cognition of innovation tolerance. This shared cognition enables teams to maintain high levels of patience and resilience when confronting uncertainties and challenges in the innovation process, persistently pursuing innovative goals and thereby enhancing the team’s overall innovation capability. Thus, it is hypothesized:

*H3:* Patient capital mediates the relationship between transformational leadership and team innovation performance through a cross-level mechanism (i.e., a 2–1-2 pathway).

**Figure 1:**
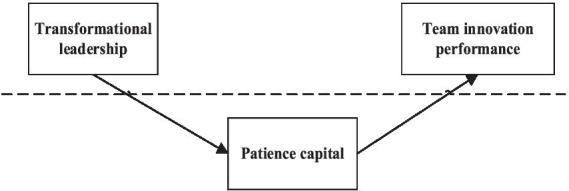
Conceptual model diagram.

The theoretical hypothesis model is illustrated in Figure 1.

## 3 Scale development and validation

### 3.1 Item development

Based on the coded data from the grounded theory analysis, an initial scale comprising 21 semantically clear measurement items was developed. To ensure the alignment of these items with the research constructs, content validity was first assessed. Experts were invited to evaluate the consistency between the initial items and the constructs. After revisions, deletions, and merging of items based on expert feedback, a consensus was reached, resulting in the retention of 16 items. Subsequently, five work teams were invited to provide feedback on the readability and accuracy of the questionnaire. They were asked to identify any ambiguities or unclear expressions, ensuring that the items were concise, clear, and unambiguous. The final scale consisted of 16 measurement items.

### 3.2 Exploratory factor analysis

Exploratory factor analysis was conducted on 242 valid questionnaires (KMO = 0.909, Bartlett’s test *p* < 0.001). Using principal component analysis with orthogonal rotation, six factors were initially extracted, accounting for 72.61% of the total variance. After removing items with low factor loadings, the final scale comprised 15 items across five factors. The remaining items exhibited factor loadings ranging from 0.753 to 0.901, with a cumulative variance explanation of 71.46%, indicating significantly improved structural validity. The results are presented in Table 6.

**Table 6:** Results of exploratory factor analysis.

Item	F1	F2	F3	F4	F5	Item-total correlation	Cronbach’s α if item deleted
Able to effectively manage emotional fluctuations	0.852					0.742***	0.908
Remain calm and make rational decisions under pressure	0.823					0.733***	0.907
Maintain focus to adhere to long-term goals	0.791					0.699***	0.910
Quickly regain motivation after facing failure		0.838				0.712***	0.911
Sustain high levels of engagement in long-term projects		0.807				0.697***	0.910
Proactively adjust strategies when encountering resistance		0.775				0.643***	0.912
Regularly review goals and refine strategies			0.784			0.725***	0.907
Translate organizational strategies into personal action guidelines			0.752			0.697***	0.911
Delay gratification for greater long-term gains			0.718			0.687***	0.909
Sensitively perceive emotional changes among team members				0.803		0.680***	0.910
Prioritize listening over rebuttal during conflicts				0.776		0.672***	0.911
Enhance team默契 through non-verbal signals				0.709		0.658***	0.913
Dynamically adjust goals based on environmental changes					0.787	0.688***	0.910
Maintain decision-making flexibility in uncertain situations					0.754	0.692***	0.911
Embrace trial-and-error and iteratively improve solutions					0.723	0.712***	0.912

*p < 0.05; **p < 0.01; ***p < 0.001 (two-tailed test).

### 3.3 Item analysis

Item analysis indicated excellent internal consistency for the 15-item Patient Capital Scale (*α* = 0.883). Deleting any item resulted in a decrease in the α coefficient, and all items showed moderate to high positive correlations with the total score (ranging from 0.600 to 0.750, *p* < 0.001), confirming the necessity and sensitivity of the item design (Henseler et al., 2015).

### 3.4 Confirmatory factor analysis

The results of confirmatory factor analysis (maximum likelihood method) are presented in Table 7. The baseline model demonstrated good fit with the data from 303 valid questionnaires (χ^2^/df = 1.654, RMSEA = 0.035, SRMR = 0.017, CFI = 0.973, TLI = 0.962) and was significantly superior to alternative models. All item loadings ranged from 0.711 to 0.923, effectively validating the construct validity and scale appropriateness of patient capital.

**Table 7:** Results of confirmatory factor analysis.

Model	χ^2^/df	RMSEA	SRMR	CFI	TLI
Five-factor baseline	1.654	0.035	0.017	0.973	0.962
Four-factor Model A	3.199	0.101	0.070	0.835	0.855
Four-factor Model B	2.768	0.071	0.054	0.890	0.871
Three-factor Model A	3.717	0.113	0.061	0.851	0.805
Three-factor Model B	3.160	0.099	0.071	0.886	0.864
Two-factor Model A	4.147	0.119	0.033	0.843	0.771
Two-factor Model B	4.298	0.100	0.037	0.821	0.798
One-factor Model	5.138	0.121	0.072	0.724	0.697

*p < 0.05; **p < 0.01; ***p < 0.001 (two-tailed test).

### 3.5 Discriminant validity test

As shown in Table 8, the correlation coefficients between dimensions ranged from 0.380 to 0.642 (squared values: 0.154–0.365), all of which were lower than the average variance extracted (AVE), indicating good discriminant validity. Additionally, both composite reliability (0.826–0.913) and AVE (0.694–0.758) met high standards, demonstrating excellent convergent validity. The dimensions maintained reasonable correlations while possessing independent discriminability, forming a cohesive yet distinct organic structure.

**Table 8:** Discriminant validity.

Dimension	1	2	3	4	5
1. Self-control & patience	0.872	0.455^***^	0.381^***^	0.527^***^	0.488^***^
2. Persistence & resilience		0.864	0.526^***^	0.451^***^	0.642^***^
3. Long-term goal drive			0.825	0.413^***^	0.562^***^
4. Emotional intelligence & relationships				0.818	0.539^***^
5. Adaptability & flexibility					0.810

*p < 0.05; **p < 0.01; ***p < 0.001 (two-tailed test). Diagonal values represent the square root of AVE; off-diagonal values are correlation coefficients between dimensions.

To further validate the discriminant validity of the five-dimensional structure of patient capital, the Heterotrait-Monotrait Ratio (HTMT) method was applied as a supplementary test. The HTMT values reflect correlations between different dimensions, with lower values indicating better discriminant validity. As shown in Table 9, all HTMT values were below the threshold of 0.85 recommended by Henseler et al. (2015), confirming that the five dimensions of patient capital exhibit strong discriminant validity and independence. Additionally, the HTMT ratio between “Adaptability & Flexibility” and “Persistence & Resilience” was 0.68, with a correlation of *r* = 0.624 (*p* < 0.001), indicating a high synergistic relationship between these two dimensions. This finding suggests that organizations should focus on cultivating both capabilities simultaneously—enhancing individuals’ persistence and resilience while strengthening their adaptability in changing environments—to improve overall levels of patient capital.

**Table 9:** Convergent validity and HTMT test results.

Dimension	CR	AVE	Factor loadings	α	Dimension comparison	HTMT ratio	90% confidence interval
1	0.913	0.758	0.78–0.88	0.841	Self-control & patience vs. Persistence & resilience	0.52	[0.47, 0.58]
2	0.876	0.712	0.71–0.84	0.825	Self-control & patience vs. Long-term goal drive	0.44	[0.39, 0.50]
3	0.901	0.694	0.69–0.87	0.817	Self-control & patience vs. Emotional intelligence & relationships	0.61	[0.55, 0.67]
4	0.859	0.712	0.71–0.88	0.866	Persistence & resilience vs. Adaptability & flexibility	0.68	[0.63, 0.73]
5	0.826	0.730	0.70–0.89	0.843	Mean HTMT across all dimensions	0.57	—

*p < 0.05; **p < 0.01; ***p < 0.001 (two-tailed test). Diagonal values represent the square root of the Average Variance Extracted (AVE), while off-diagonal values indicate correlation coefficients between dimensions.

### 3.6 Second-order confirmatory factor analysis

**Figure 2:**
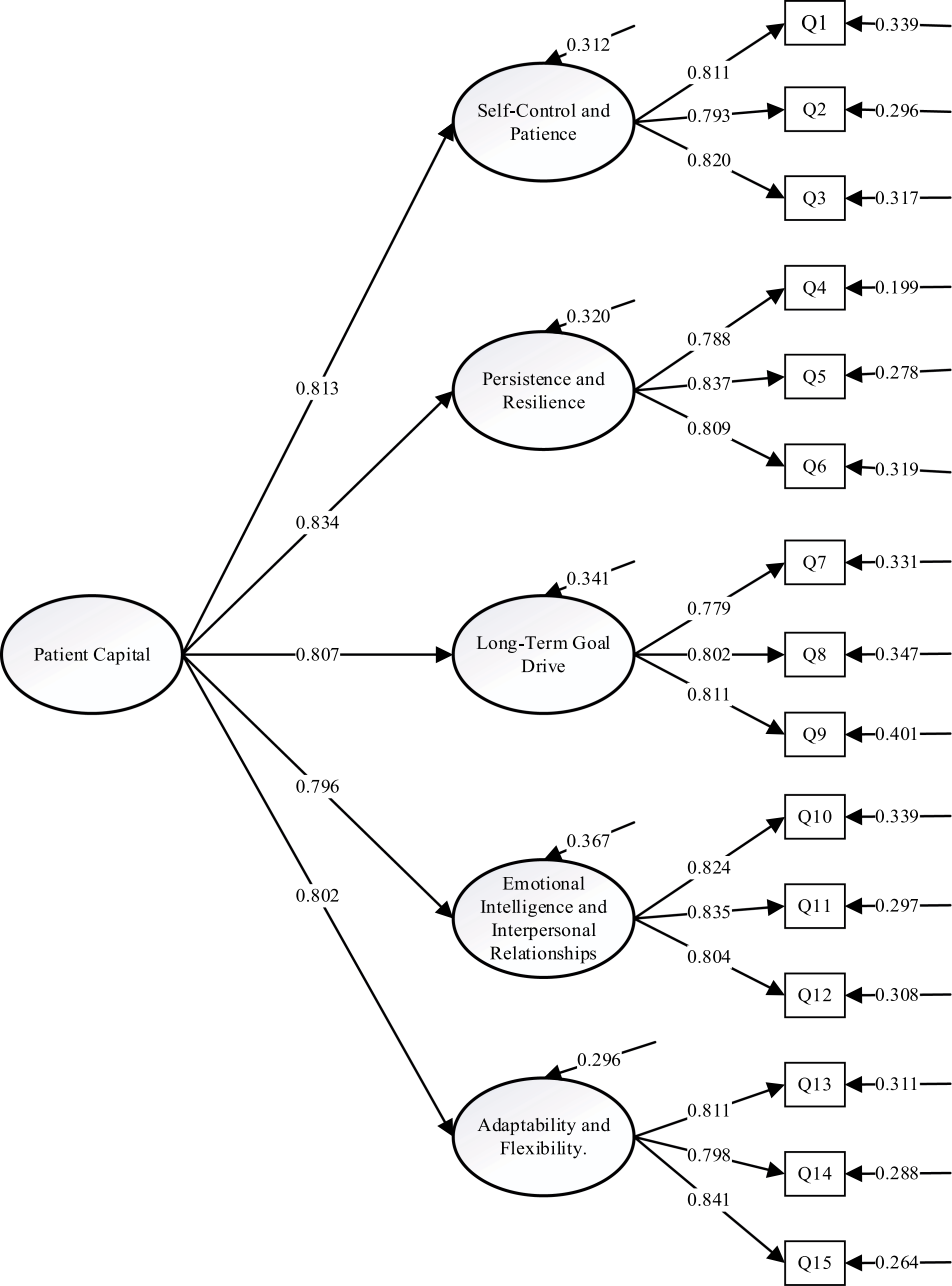
Second-order structure diagram.

The results of the second-order confirmatory factor analysis are shown in Figure 2. The standardized factor loadings were 0.813, 0.834, 0.807, 0.796, and 0.802, respectively. The model fit indices were as follows: χ^2^/df = 1.72, RMSEA = 0.038, CFI = 0.963, TLI = 0.974. All path coefficients were statistically significant (*p* < 0.001), indicating that the five dimensions effectively converge into the higher-order construct of patient capital (Fornell and Larcker, 1981).

## 4 Empirical analysis

### 4.1 Data collection

This phase employed a multi-source, multi-stage mixed data collection strategy to ensure ecological validity and statistical robustness. Specifically, the study focused on high-tech enterprises within national-level science and innovation parks, selecting 67 R&D teams as the research sample. Data collection spanned from June 2022 to December 2024 and was conducted in three stages, with intervals of more than six months between stages to effectively mitigate common method bias.

A multi-agent matching approach was adopted during data collection: leaders at various levels reported leadership behaviors and team innovation performance, while employees self-assessed their levels of patient capital. This formed a triangulated data chain encompassing “top leadership–team leadership–employees.” To ensure data quality, all responses were anonymized.

A total of 692 questionnaires were collected. After excluding responses with logical inconsistencies or more than 10% missing values, 624 valid questionnaires were retained, yielding an effective response rate of 90.2%.

### 4.2 Variable measurement

All variables were measured using a 7-point Likert scale (1 = strongly disagree, 7 = strongly agree).

#### 4.2.1 Transformational leadership

Measured using the 18-item scale developed by Wang and Howell (2010) (e.g., “My supervisor enables me to view problems from different perspectives”).

#### 4.2.2 Team innovation performance

This construct was assessed using supervisor ratings to ensure objectivity. Senior company leaders evaluated the overall innovation performance of each team using a 4-item scale developed by Lovelace et al. (2001). A sample item is: “The team’s work outcomes are highly novel.”

#### 4.2.3 Patient capital

Assessed using the 15-item scale developed in this study (e.g., “Delay gratification to achieve greater long-term gains”).

#### 4.2.4 Control variables

Based on previous literature, team tenure, team size, and demographic diversity such as age and gender may influence team innovation performance.

### 4.3 Multilevel data aggregation test

Guided by multilevel theory (Muthén and Muthén, 2012), aggregation tests were conducted for individual-level variables—transformational leadership and patient capital—that required aggregation to the team level. The results showed that the mean Rwg values were 0.89 for transformational leadership and 0.87 for patient capital, both exceeding the threshold of 0.85, indicating high within-team agreement. Furthermore, ICC1 values (0.45 for transformational leadership, 0.47 for patient capital) surpassed the significance threshold of 0.05, confirming significant between-team differences and justifying cross-level analysis. ICC2 values were both greater than 0.90, demonstrating high reliability of the aggregated data.

Team innovation performance was measured using objective team-level data (e.g., project returns, patent conversions) evaluated by the senior management team, thus not requiring aggregation tests.

### 4.4 Multilevel confirmatory factor analysis and measurement validity test

Given the limitations of Harman’s single-factor test, this study employed multilevel confirmatory factor analysis (MCFA) to examine common method bias. Unlike traditional single-level CFA, MCFA allows for independent modeling of within-group (individual) and between-group (team) measurement structures, reducing parameter estimation bias caused by level confusion. By decomposing individual variation and team means through latent group mean centering, MCFA minimizes systematic errors in common source data (Yuan and MacKinnon, 2009).

The MCFA results indicated a good model fit: χ^2^/df = 1.16, CFI = 0.98, TLI = 0.97, RMSEA = 0.017, SRMR (within) = 0.023, SRMR (between) = 0.019. All latent variable indicators were statistically significant.

### 4.5 Descriptive statistics, correlation analysis, and reliability and validity tests

Table 10 presents the descriptive statistics of the variables. The consistency tests for the transformational leadership, patient capital, and team innovation performance scales showed Cronbach’s *α* values of 0.91, 0.89, and 0.87, respectively. The composite reliability (CR) values all exceeded 0.7, indicating reliable internal consistency. Confirmatory factor analysis further demonstrated that all item loadings were greater than 0.7 (*p* < 0.001), and the average variance extracted (AVE) values for all variables were greater than the squares of their correlation coefficients, fully meeting the requirements for convergent and discriminant validity.

**Table 10:** Means, standard deviations, correlation coefficients, and reliability and validity tests of variables.

Variable	M	SD	1	2	3	4	5	6
Individual level								
1. Age	35.92	7.17						
2. Gender	0.72	0.48	0.172					
3. Tf leadership	3.92	0.85	−0.033	0.019				
4. Patient capital	3.84	0.82	0.038	0.027	0.42^***^			
Team level								
1. Leader age	47.36	6.02						
2. Leader gender	0.68	0.45	−0.47					
3. Team size	10.68	3.81	0.188	0.041				
4. Team tenure	6.52	2.64	0.063	−0.167	−0.185			
5. Tf leadership transformational leadership	3.92	0.45	−0.015	−0.133	0.078	0.091		
6. Team innovation performance	3.77	0.47	0.032	0.022	−0.212	0.184	0.313^***^	
7. Patient capital	3.84	0.60	−0.034	0.076	0.083	0.221	0.426^***^	0.522^***^

*p < 0.05; **p < 0.01; ***p < 0.001 (two-tailed test). *N* (members) = 624; *N* (teams/leaders) = 67; M = mean, SD = standard deviation; Gender: 1 = male, 0 = female.

### 4.6 MSEM results analysis

Multilevel structural equation modeling (MSEM) offers significant advantages over traditional multilevel linear modeling (MLM) (Preacher et al., 2010). First, MSEM integrates the latent variable modeling capability of structural equation modeling with the nested data processing ability of MLM, effectively separating within-group and between-group effects and avoiding parameter estimation biases caused by measurement errors or aggregation biases. Second, MSEM uses latent group mean centering to decompose individual-level variables into within-group variation and latent group means, thereby accurately identifying cross-level mediation pathways. Additionally, MSEM demonstrates superior performance in 95% confidence interval coverage for mediation effects and Type I error control (Lüdtke et al., 2008).

Therefore, Mplus 8.3 software was employed using robust full information maximum likelihood estimation (FIML-MLR) to ensure the robustness of the estimation results. The model test results are presented in Table 11.

**Table 11:** MSEM analysis results.

Path	Level relationship	Effect (β)	SE	95% CI
Direct effects				
H1: TL → PC	2 → 1	0.768^***^	0.040	[0.691, 0.846]
H2: PC → TP	1 → 2	0.321^***^	0.007	[0.308, 0.334]
Indirect effect				
H3: TL → PC → TP	2 → 1 → 2	0.247^***^	0.014	[0.218, 0.275]

*p < 0.05; **p < 0.01; ***p < 0.001 (two-tailed test). 1 = Individual level, 2 = Team level; TL = Transformational Leadership; PC = Patient Capital; TP = Team Innovation Performance; values represent unstandardized coefficients.

The MSEM results indicate that transformational leadership is positively associated with patient capital (*β* = 0.768, SE = 0.040, 95% CI [0.691, 0.846], *p* < 0.001). This finding supports transformational leadership theory and the resource-based view, suggesting that transformational leadership effectively fosters the accumulation of patient capital within organizations through vision inspiration and strategic resource commitment mechanisms. Furthermore, patient capital exerts a significant positive influence on team innovation performance (β = 0.321, SE = 0.007, 95% CI [0.308, 0.334], *p* < 0.001). Thus, Hypothesis 1 and Hypothesis 2 are supported. Bootstrap tests (5,000 samples) show that the 2–1-2 cross-level mediation effect of “transformational leadership → patient capital → team innovation performance” is significant (β = 0.247, SE = 0.014, 95% CI [0.218, 0.275], *p* < 0.001). Therefore, Hypothesis 3 is supported.

## 5 Research conclusions and discussion

### 5.1 Research conclusions

Following the qualitative research paradigm and scale development procedures of grounded theory, and utilizing NVivo 12 software combined with a Transformer architecture for coding and analyzing interview data, this study systematically deconstructed and validated a five-dimensional theoretical framework of “patient capital” in the field of human resource management. The dimensions include: self-control and patience, persistence and resilience, long-term goal drive, emotional intelligence and interpersonal relationships, and adaptability and flexibility. A definition of patient capital within the context of organizational behavior was also provided.

Based on this, multilevel structural equation modeling analysis revealed that transformational leadership is positively associated with patient capital and patient capital mediates the relationship between transformational leadership and team innovation performance.

### 5.2 Theoretical contributions

(1) This study constructs a theoretical measurement system for patient capital, effectively addressing the limitation of traditional human resource research that overemphasizes short-term performance. Traditional HR research often focuses on short-term performance indicators, such as quarterly sales and annual profits, while neglecting the psychological resources and capabilities employees accumulate over the long term. By defining and measuring patient capital, this study enriches human resource management and organizational behavior theories. Patient capital represents a critical psychological resource that enables individuals to persist toward goals, resist short-term temptations, and sustain effort in complex, uncertain, and challenging work environments.
(2) The study proposes a novel theoretical perspective by identifying patient capital as a driver of innovation. Previous innovation research has predominantly emphasized external factors such as technology, funding, and markets, paying relatively less attention to the role of individual psychological traits in fostering innovation (Xie et al., 2016). This research reveals that patient capital, as a key individual psychological trait, facilitates knowledge sharing, collaboration, and communication among team members, thereby enhancing the team’s overall innovative capability and adaptability to change. This finding provides a new theoretical lens and research framework, encouraging future studies to explore the mechanisms through which individual psychological factors influence innovation, thereby expanding the boundaries and enriching the connotation of innovation theory.
(3) The study unveils the cross-level transmission mechanism of “leadership trait → patient capital → innovative output.” While prior research has separately examined the relationship between leadership traits and team innovation, this study is the first to integrate transformational leadership with patient capital, systematically analyzing how transformational leadership is related to team innovation performance through its association with individual patient capital. This contributes to a deeper understanding of the pathways through which leadership behaviors influence organizational innovation.

### 5.3 Managerial implications

(1) Organizations should prioritize the cultivation and incentivization of patient capital. In practice, leaders can shape a long-term-oriented culture through various means, such as establishing performance evaluation systems aligned with long-term goals and avoiding excessive focus on short-term outcomes. By setting clear vision and goals, employees can see future directions and possibilities, which enhances their intrinsic motivation and commitment, thereby strengthening their resilience and perseverance in facing long-term challenges. Additionally, organizations should encourage leaders to focus on developing employees’ patient capital, particularly during complex innovation tasks, through regular communication, feedback, and guidance to help maintain long-term engagement and sustained motivation.
(2) Patient capital serves as a critical resource for driving team innovation. During prolonged technological iterations, innovation often involves multiple attempts and failures. Patient capital helps team members mitigate short-term pressures and avoid giving up due to temporary setbacks. In complex environments, patient capital enhances team members’ adaptability and flexibility, enabling them to better respond to uncertainties and changes. Therefore, organizations should emphasize the accumulation and utilization of patient capital in promoting innovation. Managers can create a supportive and inclusive work environment that encourages diverse ideas and perspectives, respects individuality and differences, and enhances psychological safety. Encouraging self-motivation and persistence in the face of difficulties, along with team-building activities and role modeling, can further boost the team’s overall innovative capability.
(3) Strengthening organizational-level patient capital management mechanisms can facilitate the effective translation of leadership efficacy into innovation performance. Organizations may design deferred incentive mechanisms, such as equity incentives and long-term performance bonuses, allowing employees to share in the long-term success of the organization, thereby enhancing their sense of belonging and loyalty. Cultivating an organizational historical memory system that records and transmits the organization’s development journey, successes, and lessons learned can help new employees better understand the organizational culture and values, strengthening cohesion and continuity.

### 5.4 Limitations and future research directions

Although this study has achieved certain results in exploring the relationships among transformational leadership, patient capital, and team innovation performance, several limitations remain. Future research could expand and deepen the inquiry in the following aspects: Finally, future research could delve deeper into the dynamic and systemic nature of patient capital. Our study establishes static, cross-sectional relationships. To understand how patient capital is accumulated, depleted, and interacts in complex systems, future work should employ longitudinal designs and borrow frameworks from other fields. For instance, competition and cooperation dynamics within or between teams regarding limited resources can be examined using evolutionary game theory models (Qiao et al., 2024). Furthermore, the concept of “risk contagion” in project setbacks or market uncertainties provides a lens to study how patient capital acts as a buffer or control mechanism to prevent the spread of negative impacts within a team or organization. Exploring patient capital through these dynamic and complex system perspectives will significantly enhance its theoretical depth and practical relevance.

## Data Availability

The datasets generated during and/or analysed during the current study are available from the corresponding author on reasonable request.
